# Transfer Learning for Sentiment Classification Using Bidirectional Encoder Representations from Transformers (BERT) Model

**DOI:** 10.3390/s23115232

**Published:** 2023-05-31

**Authors:** Ali Areshey, Hassan Mathkour

**Affiliations:** Department of Computer Science, College of Computer and Information Sciences, King Saud University, Riyadh 11543, Saudi Arabia; mathkour@ksu.edu.sa

**Keywords:** BERT model, sentiment analysis, machine learning, transformers, transfer learning

## Abstract

Sentiment is currently one of the most emerging areas of research due to the large amount of web content coming from social networking websites. Sentiment analysis is a crucial process for recommending systems for most people. Generally, the purpose of sentiment analysis is to determine an author’s attitude toward a subject or the overall tone of a document. There is a huge collection of studies that make an effort to predict how useful online reviews will be and have produced conflicting results on the efficacy of different methodologies. Furthermore, many of the current solutions employ manual feature generation and conventional shallow learning methods, which restrict generalization. As a result, the goal of this research is to develop a general approach using transfer learning by applying the “BERT (Bidirectional Encoder Representations from Transformers)”-based model. The efficiency of BERT classification is then evaluated by comparing it with similar machine learning techniques. In the experimental evaluation, the proposed model demonstrated superior performance in terms of outstanding prediction and high accuracy compared to earlier research. Comparative tests conducted on positive and negative Yelp reviews reveal that fine-tuned BERT classification performs better than other approaches. In addition, it is observed that BERT classifiers using batch size and sequence length significantly affect classification performance.

## 1. Introduction

Sentiment analysis, or opinion mining, is the computational study of people’s opinions, feelings, emotions, and attitudes toward entities such as products, services, questions, events, subjects and their attributes [1]. For example, sentiment analysis can track the mood of the public toward a particular entity in order to create actionable knowledge. In addition, this kind of knowledge may be useful in understanding, explaining, and predicting social phenomena. In the business domain, sentiment analysis plays a critical role in helping businesses improve their strategy and better understand customers’ feedback on their products. In today’s client-centric business culture, understanding the client is becoming more important. Because of the importance of sentiment analysis for business and society, it has spread from computer science to the sciences of management and social. Over the past few years, industrial activities related to sentiment analysis have also flourished: Many large businesses or organizers have built their own internal capabilities [2]. Businesses need to understand human emotions because consumers can now express themselves more freely than ever before. Products may actively listen to their customers by dynamically evaluating feedback from surveys and social media posts to create products and services that are tailored to their needs [3].The traditional sentiment analysis techniques mostly concentrate on characteristic engineering and machine learning approaches. The characteristics, comprising n-grams, bag-of-words (BoW), term frequency–inverse document frequency (TF-IDF), and part-of-speech (POS) tags, are first extracted from the text. Subsequently, classification methods, such as naïve Bayes (NB), K-nearest neighbor (KNN) and support vector machine (SVM), are applied to these characteristics to classify the sentiment’s polarity [3]. Recently, studies have focused on transfer learning. Transfer learning is a state-of-the-art deep learning method for natural language processing (NLP) challenges [4,5]. In 2018, BERT was introduced, “which stands for Bidirectional Encoder Representations from Transformers” model [4]. It is intended to jointly condition both left and right contexts in all layers in order to pre-train deep bidirectional representation from unlabeled text [4]. Based on factors such as the quantity of transformer layers, self-attention layers, word embedding, forms of fine-tuning, masking, parameters, and others, different BERT transfer learning model types exist [5]. To address this knowledge gap and deliver timely insight, this research attempts to exploit the applicability of BERT in sentiment analysis and to what extent it will improve performance as compared to traditional machine learning when employing proper preprocessing and suitable fine-tuning. We do this for text reviews that are positive as well as negative. We take advantage of the fact that the BERT tokenizer generates contextualized token embeddings instead of manually creating them. Using reviews taken from the Yelp dataset for training and testing, a BERT that has been fine-tuned for review classification is evaluated. To compare the effectiveness of BERT-based classifiers for the categorization of reviews, their performance is compared with that of machine learning techniques, such as KNN, NB, and SVM [6]. In addition, the proposed models are compared with previous studies of the same. Our work is motivated by trying to achieve better performance while keeping a simple model that permits automatic preprocessing as opposed to manual preprocessing. BERT provides an opportunity to attain that. We further aim for higher prediction accuracy via fine-tuning as compared to previous studies. The remaining parts of this paper are broken into the following sections. A short overview of existing literature is presented in Section 2. Section 3 of this article provides a description of the study’s research methodology. In Section 4, the outcomes are shown and discussed. Section 5 of this article discusses the implications of this study. Finally, the study is concluded in Section 6.

## 2. Literature Review

The growth of social media has added to the importance of the web as a source of knowledge. It has been observed that the number of people who frequently use social networking is increasing. Web reviews are one sort of user-generated content that focuses on the individual perception of a product, service, event, or subject [7]. Many approaches based on various characteristics and machine learning techniques have been presented and evaluated using various datasets, such as Amazon, Yelp, and other resources. Hemalatha et al. [8] investigated sentiment analysis using Yelp review datasets. They compared machine learning techniques, such as NB, MNB, and SVC. They illustrated that the NB using TF-IDF had the highest accuracy of 79% among all the others. Govind et al. [9] evaluated various techniques on well-known datasets, such as Yelp in an attempt to identify the most efficient techniques for sentiment mining, including Unigrams, Bigrams, SVM, NB, and random forests (RF). When the author compares the RF method to traditional methods, they discover that sentiment mining is significantly improved. Liu [10] compared how well various deep learning and machine learning models performed at predicting user sentiment. The author discovered that less complicated models, such as logistic regression (LR) and SVM, are better at predicting sentiment than more sophisticated models, such as gradient boosting (GB), LSTM, and BERT. Liu [11] studied Yelp review datasets on machine learning and transfer learning models. The machine learning models include NB, LR, RF, and SVM. In addition, the transformer models BERT, DistiBERT, RoBERTa, and XLNet are applied [12,13]. The highest accuracy of 70% was achieved by XLNet. Durairaj and Chinnalagu [14] suggested a fine-tuned BERT model to predict customer sentiment by using customer reviews from various datasets. The proposed model’s performance was compared with SVM, FastText, BiLSTM and hybrid FastText-BiLSTM models. The outcome of the experiment demonstrates that the BERT model outperforms other models. Alamoudi and Alghamdi [7] proposed machine learning, deep learning, and transfer learning-based models for the sentiment classification on the Yelp review dataset, and the ALBERT model achieved 98.30% accuracy [15]. Xu et al. [16] used BERT to evaluate online product reviews’. The BERT model is accurate in predicting reviews. Prottasha et al. [17] compared the performance of many modeling methods (Word2Vec, GloVe, FastText, and BERT) and found that a properly calibrated BERT is superior to the competition in many natural language processing (NLP) applications, especially in the area of sentiment analysis. Bilal et al. [18] suggested features for the reviewer’s network strength and adapted them to estimate helpfulness. In order to predict the efficiency of reviews, a variety of algorithms based on shallow learning were applied to around forty different parameters connected to the evaluation, and the person who wrote it. Ge et al.’s [19] proposal for recommending reviews was to have the model predict how useful a review would be based on the ratings given to those reviews that had already received positive ratings. Mutinda et al. [20] suggested the LEBERT model, which employs an n-gram to segment the input text and a sentiment lexicon to determine whether segments of the text contain sentimental words. BERT then uses these detected sections’ words to create a vector. The resulting word vector is fed into a fully connected layer in a convolutional neural network (CNN) to extract features. Overall, the proposed LeBERT model was 88.2% accurate when used for binary classification. Zhang et al. [21] presented a Sliced BI-GRU (bidirectional-gated recurrent unit) architecture that employs BERT embedding in conjunction with the multi-head self-attention mechanism. The BERT models’ word vector representation first, which plays a role in the neural network’s embedding layer, and then they divide the input sequence into equal-length chunks. Additional features are extracted with the help of Bi-sequence GRU. According to experimental results, this model achieves an impressive 74.37% accuracy in classification on the Yelp dataset. Başarslan and Kayaalp [22] employed SVM and NB techniques from machine learning as well as CNN, RNN, and LSTM techniques from deep learning to categorize the responses. BERT, Glove, Word2Vec, TF-IDF, and BOW are some of the word embedding techniques employed. The model built using BERT and LSTM proved to be the most effective of all the models tested. Out of all the text representations and word-embedding approaches, BERT proved to be the most effective when used in a model. Using the Yelp review dataset, machine learning achieved accuracy rates of 80–86% and deep learning models achieved accuracy rates of 81–89%. Benarab and Gui [23] suggested a CNN-enhanced transformer encoder to produce a more generalizable representation using convolutional layers, identify similarities between representations from all BERT layers, and to compute the average employing the multi-head attention method. On Yelp datasets, the suggested method obtains an accuracy of 82.23%. Better results are achieved when BERT is combined with Bi-LSTM, CNN, and RNN rather than Word2vec, as suggested by Bello et al. [24]. While Word2vec limits our capacity to understand the context in which a word is used, BERT takes the previous and following inputs into account. We discovered that sentiment analysis has not been adequately studied on the Yelp review dataset by reviewing the literature. There is no previous research comparing the performance of different types of prediction models, such as machine learning and transfer learning. As a result, this proposed investigation will address a research gap. The purpose of this paper is to compare the efficacy of different classification models.

## 3. Research Methodology

This section explains how to gather data, label them, split the dataset into training and testing data, fine-tune BERT, train machine learning techniques, and measure classifier performance to distinguish between positive and negative reviews.

### 3.1. Data Collection and Labeling

Yelp review datasets are employed in this study to identify positive and negative reviews. Yelp is an established platform for crowd-sourced reviews and ratings that was launched in 2004 [25]. Review texts for thousands of samples are included in it [26]. There are also plenty of data about businesses, reviews, and users in the Yelp Open Dataset [27,28]. The Yelp dataset has been shown to be useful for scientific, educational, and personal uses [11]. Reviews that receive four or more positive votes are considered positive, while reviews that receive none at all are considered negative. According to the existing literature, Ge et al. [19] and Bilal et al. [18] discarded all of the reviews, with the exception of those with a rating of 0 or 4, which respectively reflect negative and positive reviews. In total, 10,000 reviews are chosen from the used dataset, employing stratified sampling methods [18]. The resulting dataset consists of 5000 records each for positive and negative reviews. The total number of sentences is 10,000, with a total of 740,877 tokens. The average number of tokens per sentence is 75, and the positive-to-negative sentiment ratio is 1.0.

### 3.2. Dataset

In this research, a dataset of 10,000 Yelp reviews with an equal balance of positive and negative reviews is used. The stratified sample method is used to split the dataset into training and testing datasets. Training comprises 80% (8000 samples) of the Yelp dataset, and testing comprises the remaining 20% (2000 samples). Ten percent of BERT’s training dataset samples was used for validation through each training cycle. The Yelp dataset used in this research is explained in detail in Table 1.

### 3.3. Fine-Tuning BERT

On a variety of NLP challenges, such as text classification, autocomplete or autosuggest, question answering, etc., BERT has shown cutting-edge performance. Employing BERT also has the advantages of rapid development, fewer data requirements, and improved outcomes [4]. The fine-tuning of BERT for text classification is based on the following steps: The BERT model requires a particular structure for the input data in order to be trained. In the beginning, the data (review text) are tokenized with the specific BERT model for tokenization; in this research, the BERT-based uncased model is used. The BERT tokenizer is employed to tokenize the text. For example, the input text is tokenized as shown: Input text: [‘Wonderful service Very clean restaurant Food was fantastic Definitely a permanent customer’]; Tokenized: [‘wonderful’, ‘service’, ‘very’, ‘clean’, ‘restaurant’, ‘food’, ‘was’, ‘fantastic’, ‘definitely’, ‘a’, ‘permanent’, ‘customer’]. After word tokens are created, at the front of the text, a special [CLS] token is appended, and at the end of the text, a special [SEP] token is appended. Special Tokens: [‘[CLS]’, ‘wonderful’, ‘service’, ‘very’, ‘clean’, ‘restaurant’, ‘food’, ‘was’, ‘fantastic’, ‘definitely’, ‘a’, ‘permanent’, ‘customer’, ‘[SEP]’]. In the next steps, based on the tokenizer vocabulary, the produced tokens are instead mapped to their corresponding indexes, as follows: Tokens IDs: [101, 6919, 2326, 2200, 4550, 4825, 2833, 2001, 10392, 5791, 1037, 4568, 8013, 102]. The experimentation in this research uses different sequence lengths: 64, 128, 320, 384, and 512. After determining the length of the sequence, which can be between 64 and 512, all of the reviews are either extended until they reach the desired size or shortened based on that size. To distinguish the different tokens from padding tokens, attention masks are appended at the end. The following example uses a sequence length of 64 to illustrate these steps: input_word_ids: [101, 6919, 2326, 2200, 4550, 4825, 2833, 2001, 10392, 5791, 1037, 4568, 8013, 102, 0, 0, 0, 0, 0, 0, 0, 0, 0, 0, 0, 0, 0,0, 0, 0, 0, 0, 0, 0, 0, 0, 0, 0, 0, 0, 0, 0, 0, 0, 0, 0, 0, 0, 0, 0, 0, 0, 0, 0, 0, 0, 0, 0, 0, 0, 0, 0, 0, 0] input_mask: [1, 1, 1, 1, 1, 1, 1, 1, 1, 1, 1, 1, 1, 1, 0, 0, 0, 0, 0, 0, 0, 0, 0, 0, 0, 0, 0, 0, 0, 0, 0, 0, 0, 0, 0, 0, 0, 0, 0, 0, 0, 0, 0, 0, 0, 0, 0, 0, 0, 0, 0, 0, 0, 0, 0, 0, 0, 0, 0, 0, 0, 0, 0, 0] input_type_ids: [0, 0, 0, 0, 0, 0, 0, 0, 0, 0, 0, 0, 0, 0, 0, 0, 0, 0, 0, 0, 0, 0, 0, 0, 0, 0, 0, 0, 0, 0, 0, 0, 0, 0, 0, 0, 0, 0, 0, 0, 0, 0, 0, 0, 0, 0, 0, 0, 0, 0, 0, 0, 0, 0, 0, 0, 0, 0, 0, 0, 0, 0, 0, 0]. The tensor dataset is created for BERT classifier model training using input word IDs, input masks, and labels from the Yelp review dataset.

**Figure 1:**
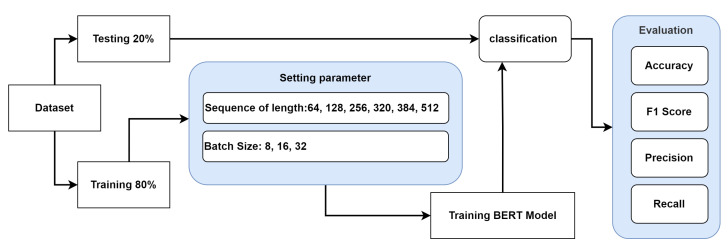
Transfer learning (BERT) diagram model.

Training comprises 80% of the Yelp dataset, and testing comprises the remaining 20%. Testing involves 2000 samples, while training involves 8000 samples. Following that, the training dataset is divided into training (90% of it) with 7200 samples and testing (10% of it) with 800 samples. The BERT model consists of 12 transformer blocks, 12 self-attention heads, and 768 hidden sizes. In this research, we fine-tune BERT for the classification tasks using Google Colab. BERT classification models are built using all lengths of sequence, 64, 128, 256, and 320, with all batch sizes set to 32 for training as well as testing. In addition, the BERT classification model is constructed with a sequence of length 384 and a batch size of either 16 or 32, depending on whether it is being used for training as well as testing. Additionally, the BERT classification is built with a sequence of length 512 and a batch size of either 8 or 32 for training as well as testing. The researchers suggest 16 and 32 batch sizes for fine-tuning BERT [4]. Table 2 provides the BERT base model’s hyper-parameters. Each batch inside an epoch requires a new set of parameters, which the algorithms must update. The proposed approach for the BERT model is depicted in Figure 1. In order to evaluate the efficacy of each training iteration, researchers employ a validation split to compute validation loss and accuracy. In this study, various classifiers are fine-tuned based on various sequence lengths.

### 3.4. Machine Learning Approaches

**Figure 2:**
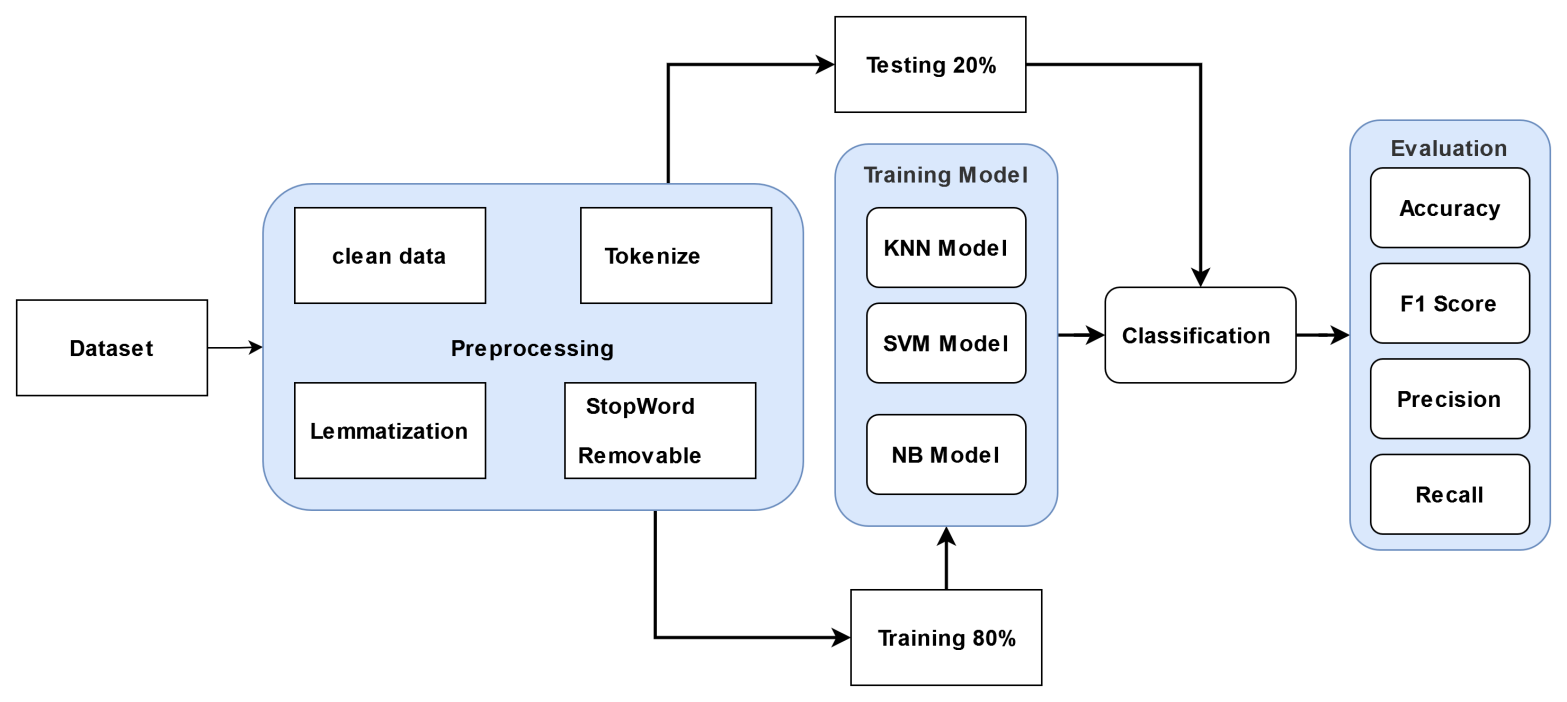
Machine learning diagram model.

In this research, the efficiency of the fine-tuned BERT-based model cannot be determined without comparison to non-BERT models. In order to classify positive and negative reviews, text classifiers KNN, NB, and SVM are trained. Textual features are generated from text using the term frequency–inverse document frequency (TF-IDF), and then machine learning models (KNN, NB, and SVM) are trained. Table 3 provides the machine learning model hyper-parameters. Clean data, tokenization, stop word elimination, and lemmatizing techniques are used for preprocessing methods. On the basis of TF-IDF, word vectors are produced. Figure 2 shows the steps of the proposed methodology for machine learning methods.To ensure that the machine learning model is constructed effectively and efficiently, preprocessing focuses on removing irrelevant characteristics and extracting relevant ones from the text. The following procedures were used to preprocess Yelp review datasets:

1. The term “clean data” refers to the practice of deleting extraneous characters from text, such as HTML tags and trailing letters after apostrophes (such as the s in it’s). Additionally, take off the web address and any punctuation. Moreover, replace all non-alphanumeric characters with a single space. For instance, “123&%, Me, 2 2023” would become “123 Me 2 2023”. These symbols may cause noise in the data because they do not have much meaning. Thus, symbols are eliminated from the Yelp reviews [29]. Such a process does not impact the semantics of the dataset.
2. Tokenization is a method of breaking down a string of characters into individual words. A token’s value is not dependent on any other tokens [29].
3. The stop words are eliminated because they do not considerably improve the clarity of the data. Eliminating stop words shifts the focus to meaningful words, which reduces the text’s dimensionality and makes it easier to discern patterns and meanings.
4. Lemmatization: The process of “lemmatizing” a set of inflected words into their standard form (the “lemma”) is a common natural language processing (NLP) process. The following is an example: kites becomes kite, corpora becomes corpus, feet becomes foot, etc. [30].

For example, in the input text [“Wonderful service Very clean restaurant &$\hat{\%}$ Food was fantastic Definitely a permanent customer ”], the input text is used for the clean process and becomes [“Wonderful service Very clean restaurant Food was fantastic Definitely a permanent customer”]. After that, the text is tokenized [“Wonderful”, “service”, “Very”, “clean”, “restaurant”, “Food”, “was”, “fantastic”, “Definitely”, “a”, “permanent”, “customer”]. In the next step, the stop words are eliminated from the tokens [“Wonderful”, “service”, “Very”, “clean”, “restaurant”, “Food”, “fantastic”, “Definitely”, “permanent”, “customer”], then limmatization is applied [“Wonderful”, “service”, “Very”, “clean”, “restaurant”, “Food”, “fantastic”, “Definitely”, “permanent”, “customer”]. As it can be seen, such a process does not cause a loss of data.

### 3.5. Performance Evaluation

In order to predict positive reviews, a binary classification task is used, with positive reviews being labeled as true (1) and negative reviews being labeled as false (0). Both the BERT- and machine learning-based classifiers are tested on a testing dataset containing 2000 instances to determine how well they perform. After passing through the initial formatting procedure, the test data are transformed into formats suitable for use with BERT. A few preprocessing methods are applied to generate word vectors before testing KNN, NB, and SVM. The sequence lengths employed for BERT classifier evaluation are identical to those employed for fine-tuning the corresponding BERT-based model. As an example, the length of sequence 64 is used in both the fine-tuning and evaluation of a BERT model. The literature uses a variety of different classification models and evaluation metrics. As a result, the problem domain and the dataset’s attributes, such as balance or imbalance, should be taken into account when choosing the right metrics. Accuracy, precision, recall, and F1 score are used in this research as measurement methods to measure the classifier performance. Eventually, by comparing the fine-tuned BERT model to the KNN, NB, and SVM, its performance in classifying positive and negative reviews is evaluated.

## 4. Result and Discussion

**Figure 3:**
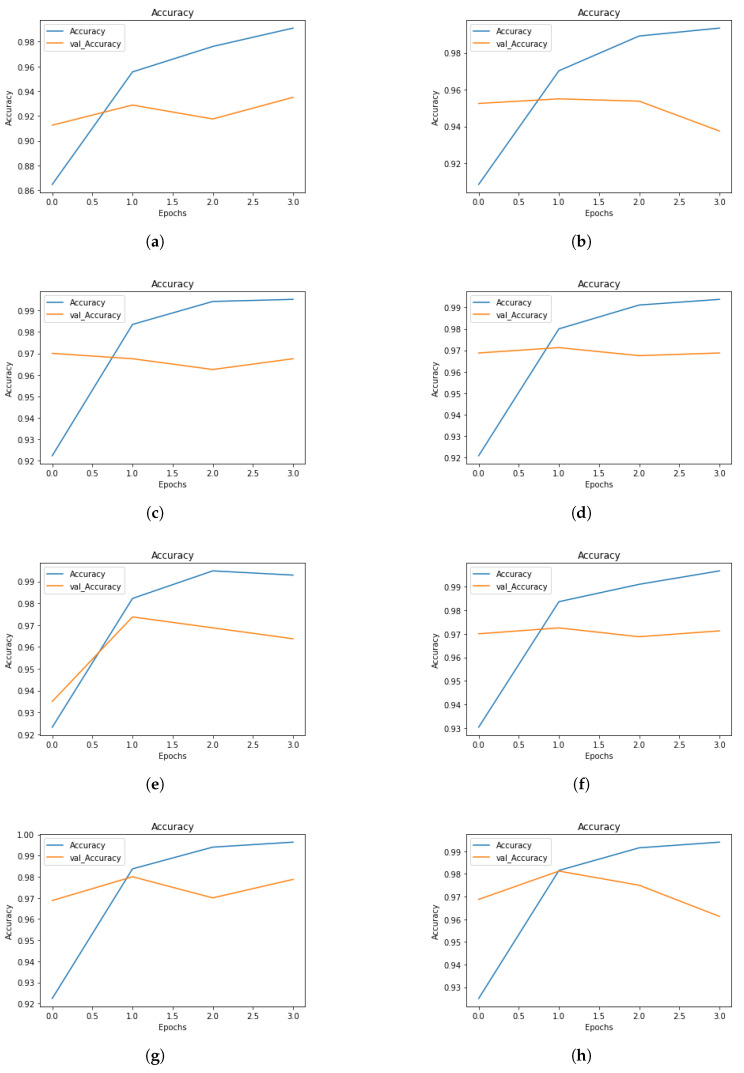
Different batch sizes and sequence lengths for training and validation loss. (**a**) Sequence length: 64, batch size: 32. (**b**) Sequence length: 128, batch size: 32. (**c**) Sequence length: 256, batch size: 32. (**d**) Sequence length: 320, batch size: 32. (**e**) Sequence length: 384, batch size: 16. (**f**) Sequence length: 512, batch size: 8. (**g**) Sequence length: 384, batch size: 32. (**h**) Sequence length: 512, batch size: 32.

The evaluation outcomes of various fine-tuned BERT models classified using various sequence lengths are presented and addressed in this part. To further examine and evaluate the efficacy of the various methodologies employed in this work, the evaluation outcomes for machine learning-based classifiers (KNN, NB, and SVM) are presented and addressed. The overall outcome of fine-tuning BERT classification models for the various sequence lengths used in this experiment is presented in Table 4 for training and validation. The results comprise training time, batch size, training loss, validation loss, training accuracy, and validation accuracy for four epochs for each length sequence. Losses during training and validation for the BERT classification model are depicted in Figure 3 for the following lengths of sequence: 64, 128, 256, 320, 384, and 512. The BERT-based model is initially trained and validated using the length of sequence 64 to ensure optimal performance. Figure 3a depicts the training and validation losses for a classifier using batch 32 with a length of sequence 64. From 0.301 in epoch 1 to 0.026 in epoch 4, the training loss is reduced. In contrast to the highest validation accuracy achieved in epoch 4, from epoch 1 to epoch 4, the validation loss increases from 0.214 in epoch 1 to 0.350. The continuously rising validation loss indicates that more training will result in overfitting.

The training and validation loss outcomes of a BERT classification model with a length of sequence of 128 using a 32 batch size shows in Figure 3b that training loss went from being 0.224 in epoch 1 to being 0.023 in epoch 4, and similarly, the validation loss went down from 0.169 in epoch 1 to 0.158 in epoch 2. After that, in epoch 3, the validation loss grew to 0.188 and to 0.302 in epoch 4. Epoch 2 had the highest validation accuracy. The outcomes of a BERT classification model trained with length of sequence 256 and a batch size set to 32 are shown in Figure 3c. Training loss was 0.182 in epoch 1, and it dropped to 0.016 by the end of epoch 4. On the other hand, between epoch 1 and 4, the validation loss jumped from 0.098 to 0.127. In epoch 2, the highest validation accuracy was achieved. Figure 3d shows four epochs of a BERT classification model trained with a length of sequence 320 and batch size 32. Training loss was shown to reduce from 0.192 in epoch 1 to 0.021 in epoch 4. In contrast, the validation loss increased from 0.103 in epoch 1 to 0.149 in epoch 3, then decreased to 0.112 in epoch 4. The best validation accuracy was obtained in epoch 2.

Figure 3e displays the training and validation loss measures for a BERT classification model with a length of sequence 384 and batch size 16. The training loss decreased from 0.179 in epoch 1 to 0.018 in epoch 3, then increased to 0.021 in epoch 4. In addition, after starting at 0.203 in epoch 1, the validation loss dropped to 0.104 by epoch 2, then rose to 0.145 by epoch 4. The highest validation accuracy was achieved in epoch 2. Data for a BERT classification model with a length of sequence 512 and batch size 8 are shown in Figure 3f.

The training loss decreased from 0.169 in epoch 1 to 0.013 in epoch 4. However, the training loss increased from 0.097 in epoch 1 to 0.134 in epoch 4. The best validation accuracy was obtained in epoch 2. Figure 3g shows 4 epochs of a BERT classification model trained with a length of sequence 384 and batch size 32. The best validation accuracy was obtained in epoch 2. The training loss decreased from 0.180 in epoch 1 to 0.012 in epoch 4. Furthermore, the validation loss decreased from 0.101 in epoch 1 to 0.090 in epoch 2, then increased to 0.123 in epoch 3, followed by a decrease to 0.105 in epoch 4. The results of a BERT classification model with a length of sequence 512 and a batch size 32 are displayed in Figure 3h.

In epoch 2, the highest validation accuracy was reached. The training loss decreased from 0.186 in epoch 1 to 0.019 in epoch 4. In contrast, the validation loss decreased from 0.105 in epoch 1 to 0.085 in epoch 2, and then increased to 0.159 in epoch 4. Classifiers with different sequence lengths and batch sizes are compared. The training loss is shown to diminish steadily over the period of 4 iterations. In contrast, there is no pattern to the validation loss, which is totally random. Additionally, the validation accuracy tends to vary randomly. The highest validation accuracy (0.981) is achieved with a length of sequence 512 and a batch size 32. Conversely, the worst validation accuracy (0.935) is achieved with a length of sequence 64 and batch size 32.

**Figure 4:**
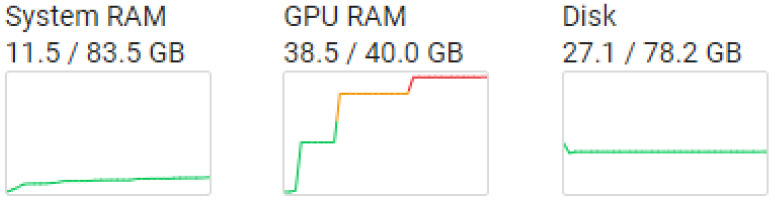
Google Colab resources.

The research shows that training and validation times grow exponentially with sequence length. Classifier training and validation times vary with sequence and batch sizes, as well as the automatically assigned GPU performance in Google Colab. The experiments were carried out in Python on a Google Colab Jupiter notebook with NVIDIA A100-SXM, 83.5 GB of memory, and a 40 GB GPU, as shown in Figure 4. The BERT classifier required 11.5 GB of memory and 38.5 GB of GPU to run with a batch size 32 and a maximum length of sequence 512.

In addition to training several BERT models, the dataset of 8000 samples is used to train KNN, NB, and SVM classification models based on TF-IDF. Then, a test dataset consisting of 2000 reviews is used to evaluate the efficacy of KNN, NB, SVM, and different BERT classifiers. Table 5 provides an overview of the prediction outcomes for each classifier used in this research, with the abbreviations TP, FP, FN, and TN standing for true positive, false positive, false negative, and false positive, respectively. The evaluation’s findings indicate that KNN predicts 867 TP, 158 FP, 133 FN, and 842 TN. 972 TP, 104 FP, 73 FN, and 896 TN are predicted by the NB. SVM, on the other hand, predicts 959 TP, 68 FP, 41 FN, and 932 TN.

The results show that with a length of sequence 64 and a batch size 32, the BERT classification model can accurately predict 907 TP and 933 TN samples. However, it predicts 82 FN and 62 FP samples. Based on target label comparison, the BERT classification model with length of sequence 128 and batch size 32 predicts 924 TP samples, 967 TN samples, 28 FP samples, and 65 FN samples. In total, 949 TP samples, 967 TN samples, 28 FP samples, and 40 FN samples are produced by the predictions of the BERT classifier with a sequence length of 256 using batch size 32. In addition, the BERT classification model with a length of sequence 320 and using batch size 32 yields a prediction of 973 TP samples, 948 TN samples, 47 FP, and 16 FN samples, respectively.

In total, 967 TP samples, 960 TN samples, 35 FP samples, and 22 FN samples are produced by the predictions of the BERT classifier with a sequence length of 384 using batch size 16. The BERT classification model results with a length of sequence 512 using batch size 8 show, from the predictions, 952 TP, 973 TN, 22 FP, and 37 FN.Additionally, 965 TP samples, 966 TN samples, 29 FP samples, and 24 FN samples are produced by the predictions of the BERT classification model using batch size 32 with a length of sequence 384. Finally, the BERT classification model using batch size 32 and length of sequence 512 predicts 936 TP and 984 TN, compared to 11 FP and 53 FN.

Table 6 shows the results of all of the various classification models’ ratings, including accuracy, F1 score, precision, and recall. These metrics were determined using the prediction results shown in Table 5. Classifiers were compared based on their ability to sort machine learning into classification; KNN was found to have the worst accuracy (0.855) and F1 score (0.853). The F1 score of 0.910 and accuracy of 0.911 shows that NB is of higher quality than KNN. With an accuracy of (0.946) and F1 score of (0.945), SVM performed better than KNN and NB. Based on the BERT classification model results, the classification algorithm with a length of sequence 64 and a batch size 32 had the worst accuracy (0.927) and F1 score (0.926). The classifier, on the other hand, achieved the highest accuracy (0.973) and F1 score (0.973) with a length of sequence 384 and using batch size 32.

Another thing that is apparent is that a length of sequence 384 using batch size 16 and a length of sequence 512 using batch size 8 produce results that are competitively better than a sequence of length 256 using batch size 32, a sequence of length 320 using batch size 32, and a sequence of length 512 using batch size 32 with regard to performance. The findings demonstrate that the BERT-based models classification performance is significantly influenced by the sequence length and batch size used to optimize and evaluate the model.

**Figure 5:**
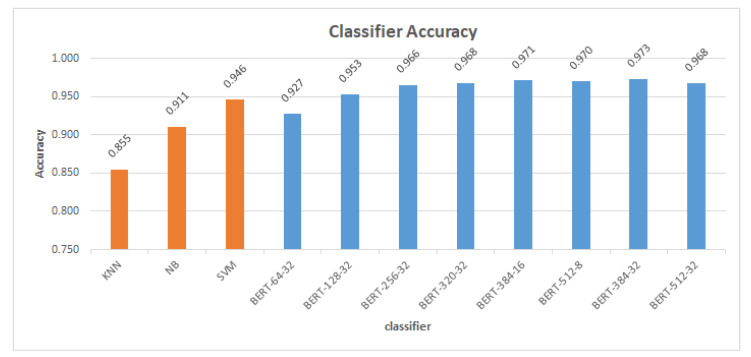
A comparison of the efficiency of machine learning and BERT classifiers.

**Figure 6:**
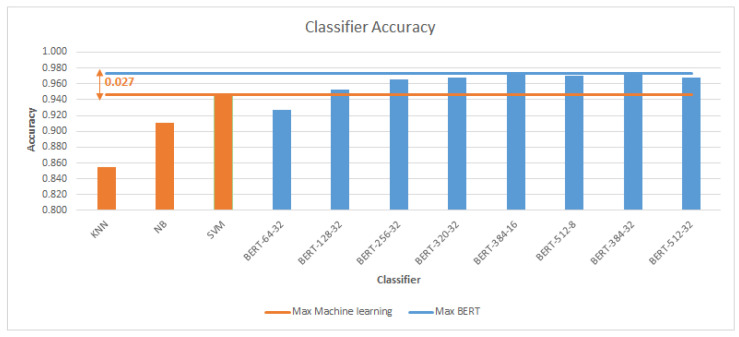
The top accuracy classifiers.

Figure 5 compares the accuracy of machine learning classifiers and BERT classifiers. According to the comparison, BERT classifiers perform better than machine learning classifiers. SVM outperforms other machine learning (KNN and NB) classifiers in terms of accuracy and F1 score. The accuracy and F1 score of SVM is higher than those of BERT with a sequence length of 64 and batch size 32. The best accuracy of 0.973% was obtained by the BERT classification model with batch size 32 and length of sequence 384 as shown in Figure 6. This is an improvement of 0.027 (2.7%) over the accuracy obtained by the SVM classifier (0.946).

After comparing the SVM and BERT prediction results in Table 5 for a length of sequence 384 and a batch size of 32, it is evident that the variation in accuracy is related to the TP, FP, FN, and TN predictions. SVM predicted 959 as TP, 68 as FP, 41 as FN, and 932 as TN, whereas BERT-384-32 predicted 965 as TP, 29 as FP, 24 as FN, and 966 as TN. Both the BERT features and sequence length with batch size used to optimize and evaluate the classifier are responsible for the effective performance of BERT-384-32. BERT features the ability to record word context in both directions without eliminating stop words, in contrast to machine learning, which omits the majority of words and does not account for word placement in context.

Table 7 displays, from the published literature of the Bilal and Almazroi models [6], various measures of categorization algorithm performance. It is worth noting that the BERT model achieved the best results compared to the others. BERT-3320-32 (0.707% accuracy) is followed by BERT-512-8 (0.697%), BERT-128-32 (0.694%), BERT-384-16 (0.683%), SVM (0.679%), BERT-64-32 (0.668%), and NB ( 0.596%) accuracy.

**Figure 7:**
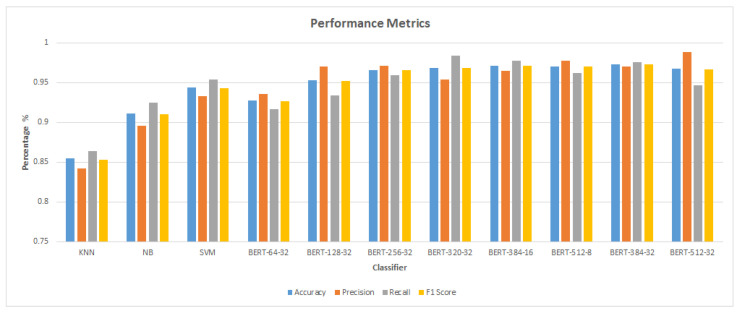
Performance metrics for the proposed models.

**Figure 8:**
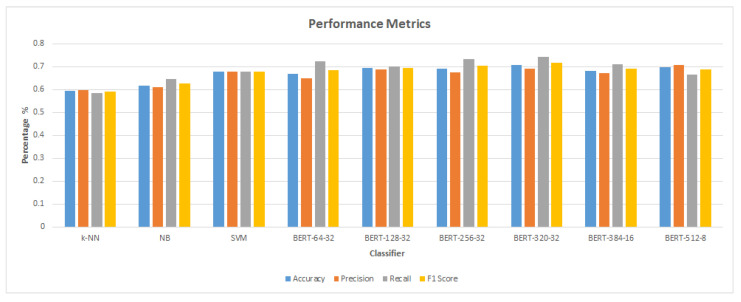
Performance metrics for Bilal and Almazroi [6] models.

Figure 7 and Figure 8 demonstrate that the suggested approach yields superior outcomes compared to the Bilal and Almazroi models [6]. Table 8 displays the results of multiple classifier models’ accuracy. It is notable that the BERT model outperformed the other models. BERT-384-32 (0.973% accuracy), is followed by BERT-384-16 (0.971%), BERT-512-8 (0.97%), BERT-512-32 and BERT-320-32 (0.968%), BERT-256-32 (0.966%), BERT-128-32 (0.953%), SVM (0.946%), BERT-64-32 (0.927%), NB( 0.911%), and KNN (0.855%), and then other models in Bilal and Almazroi [6].

**Figure 9:**
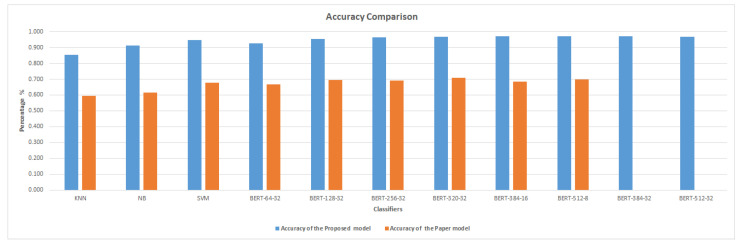
Accuracy comparison between the proposed model and Bilal and Almazroi [6] models.

The accuracy of the proposed model compared to other models in the literature is displayed in Figure 9. There is an overall enhancement to all classifier models. BERT-384-16 and NB are improved by 29%, followed by SVM, BERT-256-32, and BERT-512-8, which are improved by 27%. KNN, BERT-64-32, BERT-128-32 and BERT-320-32 have improved accuracy of 26%.

**Figure 10:**
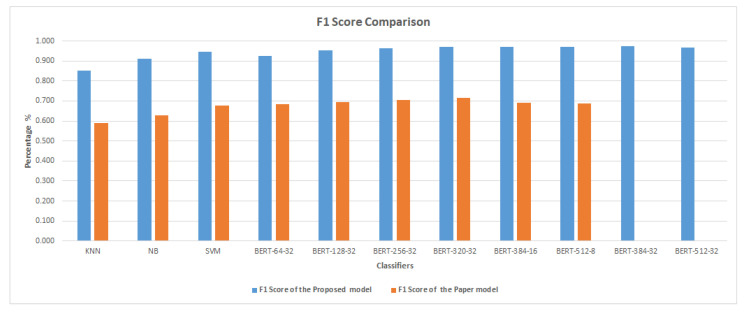
F1 score comparison between the proposed model and Bilal and Almazroi [6] models.

Table 9 shows the F1 score for classifier models for the proposed model and Bilal and Almazroi models [6]. It is notable that the BERT model outperformed the other models with the highest F1 score. BERT-384-32 (0.973% F1 score) is followed by BERT-384-16 (0.971%), BERT-512-8 (0.97%), BERT-320-32 (0.969%), BERT-512-32 (0.967%), BERT-256-32 (0.965%), BERT-128-32 (0.952%), SVM (0.945%), BERT-64-32 (0.926%), NB (0.91%), and KNN (0.853%), and then other models in [6]. Figure 10 shows the proposed model’s F1 score in comparison to other models found in the literature. There is an overall enhancement in F1 scores for all classifier models. BERT-512-8, BERT-384-16, and NB are improved by 28%, followed by SVM, which is improved by 27%. KNN, BERT-128-32 and BERT-257-32 are improved by 26%. BERT-320-32 is improved by 25%, and BERT-64-32 is improved by 24%.

Table 10 shows the accuracy of classifier models for the proposed model and previous studies on the Yelp dataset (binary label). It is notable that the proposed BERT-384-32 model outperformed the other models with the highest accuracy. The proposed BERT-384-32 model (97.3% accuracy) is followed by the proposed BERT-320-32 model (96.9%), and then LoBERT (BERT+ CNN) proposed by Mutinda et al. [20]. After that follows the BERT model proposed by Mutinda et al. [20] (84.00% accuracy) and the BERT-320-32 proposed by Bilal and Almazroi [6] (71.7% accuracy).

It is worth nothing that despite the better accuracy produced by SVM as compared to KNN and NB, SVM performs efficiently, which conforms to the finding in [31]. Unlike the finding in [31], our study shows that BERT outperforms SVM in accuracy. It is also important to note that in order to produce word vectors with the best possible quality for traditional machine learning classifiers, text data preprocessing is essential. Machine learning approaches also have the disadvantage of producing a large number of features, from which the most important ones must be selected automatically through methods for choosing features. In contrast, BERT employs the BERT tokenizer to transform content directly into a designated input structure without needing any pre-processing. On top of that, unlike machine learning methods, BERT uses a bidirectional transformer that considers both context directions.

## 5. Implications

Several theoretical and practical consequences derive from this study’s results. This research helps researchers resolve conflicting results from prior research regarding the efficiency of BERT classification models to predict accuracy by comparing the BERT model’s performance to that of machine learning approaches. Experimental data and the comparison of outcomes of the BERT classification model with machine learning techniques will also be useful for investigators in selecting the best strategy. According to the structure of the dataset used for this research, the negative reviews tend to be far longer on average than the positive ones. That will instruct you on how to create insightful evaluations using roughly 150 words. Just optimizing BERT using batch size and a specific sequence length was the focus of previous studies. In order to improve the BERT model, different batch sizes with different sequence lengths were employed in this research. These insights will allow researchers to better comprehend and evaluate the importance and influence of using various batch sizes with various sequence lengths on classifier predictive accuracy. In addition, the BERT classifier scored best when tuned using batch size 32 with a length of sequence 384, demonstrating to academics and practitioners that shortening reviews to a length of sequence 384 and using them as a prediction tool yields excellent results. However, it could change based on how the dataset is organized. Researchers will be better able to comprehend and enhance the research being conducted to predict the impact of reviews with the help of the generic technique provided in this investigation; no preprocessing was performed.

## 6. Conclusions

Online reviews are becoming more prevalent, exceeding people’s capacity to organize them in a way that is useful for making decisions about purchases. The purpose of this research is to avoid the restrictions placed on the generalizability of the solution by earlier studies on manual features, as a result, estimating the effectiveness of reviews on the internet without depending on any manually generated features. This work uses BERT, a cutting-edge method for a variety of NLP challenges, together with machine learning classifiers KNN, NB, and SVM. Using a dataset of Yelp reviews, the effectiveness of different BERT classifiers that were given varying sequence lengths to train with was evaluated and compared with the efficiency of machine learning classifiers. The evaluation’s findings demonstrated that, when it came to categorizing positive and negative reviews, tuned BERT classifiers outperformed machine learning methods. The proposed model showed improved accuracy on both accuracy and F1 scores compared with previous studies. Moreover, the maximum accuracy as well as the highest F1 score are obtained by the BERT classifier using batch size 32 with a sequence length of 384. In future work, the BERT transformer model will be fine-tuned and compared to other transformer models, such as the ALBERT, RoBERTa, and XLNet for binary classification. This study makes a contribution by investigating how accurately the BERT base model can predict the efficacy of reviews by varying the sequence lengths that it uses for its measurement. This will shed light on how reducing the length of the text (review) to an optimal length can enhance its predictive performance for researchers.

## Figures and Tables

**Table 1:** Description of datasets.

Dataset	Size	Class	Max Length	Min Length	Avg Length
Train	4000	Positive (1)	-	-	-
	4000	Negative (0)	-	-	-
	8000	Both	992	1	130
Test	1000	Positive (1)	-	-	-
	1000	Negative (0)	-	-	-
	2000	Both	958	1	129
Overall	5000	Positive (1)	941	1	104
	5000	Negative (0)	979	1	150
	10,000	Both	979	1	127

**Table 2:** Hyper-parameter tuning BERT.

Sequence Length	Batch Size	Epochs	Learning Rate
64	32	4	2.00 × 10^−5^
128	32		
256	32		
320	32		
384	16		
512	8		
384	32		
512	32		

**Table 3:** Machine learning model hyper-parameters.

Algorithm	Hyper-Parameter	Value
Naive Bayes	Smoothing Parameter	1
Support Vector Machine (SVM)	C	1.0
	Gamma	Scale
	Kernel	Radial Basis Function (RBF)
K-Nearest Neighbors (KNN)	Algorithm	Brute
	Number of neighbors	30
	Weights	Uniform
	Metric	Euclidean

**Table 4:** BERT classifier training and validation outcomes.

Sequence Length	Batch Size	Epoch	Training Loss	Training Accuracy	Valid. Loss	Valid. Accuracy
64	32	1	0.301	0.865	0.214	0.913
		2	0.124	0.956	0.231	0.929
		3	0.066	0.976	0.288	0.918
		4	0.026	0.991	0.350	0.935
128	32	1	0.224	0.909	0.169	0.953
		2	0.088	0.970	0.158	0.955
		3	0.038	0.989	0.188	0.954
		4	0.023	0.994	0.302	0.938
256	32	1	0.182	0.922	0.098	0.970
		2	0.052	0.984	0.117	0.968
		3	0.020	0.994	0.163	0.963
		4	0.016	0.995	0.127	0.968
320	32	1	0.192	0.921	0.103	0.969
		2	0.060	0.980	0.127	0.971
		3	0.030	0.991	0.149	0.968
		4	0.021	0.994	0.112	0.969
384	16	1	0.179	0.923	0.203	0.935
		2	0.054	0.982	0.104	0.974
		3	0.018	0.995	0.136	0.969
		4	0.021	0.993	0.145	0.964
512	8	1	0.169	0.930	0.097	0.970
		2	0.048	0.984	0.107	0.973
		3	0.029	0.991	0.138	0.969
		4	0.013	0.997	0.134	0.971
384	32	1	0.180	0.922	0.101	0.969
		2	0.049	0.984	0.090	0.980
		3	0.019	0.994	0.123	0.970
		4	0.012	0.996	0.105	0.979
512	32	1	0.186	0.925	0.105	0.969
		2	0.055	0.982	0.085	0.981
		3	0.028	0.992	0.114	0.975
		4	0.019	0.994	0.159	0.961

**Table 5:** A summary of the test dataset prediction results.

Classifier	TP	FN	TN	FP
KNN	867	133	842	158
NB	927	73	896	104
SVM	959	41	932	68
BERT-64-32	907	82	933	62
BERT-128-32	924	65	967	28
BERT-256-32	949	40	967	28
BERT-320-32	973	16	948	47
BERT-384-16	967	22	960	35
BERT-512-8	952	37	973	22
BERT-384-32	965	24	966	29
BERT-512-32	936	53	984	11

**Table 6:** Evaluation of classification model using the test dataset.

Classifier	Accuracy	Precision	Recall	F1 Score
KNN	0.855	0.842	0.864	0.853
NB	0.911	0.896	0.925	0.910
SVM	0.946	0.932	0.958	0.945
BERT-64-32	0.927	0.936	0.917	0.926
BERT-128-32	0.953	0.971	0.934	0.952
BERT-256-32	0.966	0.971	0.960	0.965
BERT-320-32	0.968	0.954	0.984	0.969
BERT-384-16	0.971	0.965	0.978	0.971
BERT-512-8	0.970	0.977	0.963	0.970
BERT-384-32	0.973	0.971	0.976	0.973
BERT-512-32	0.968	0.988	0.946	0.967

**Table 7:** Evaluation of classification using the Bilal and Almazroi models [6].

Classifier	Accuracy	Precision	Recall	F1 Score
KNN	0.596	0.598	0.584	0.591
NB	0.617	0.611	0.645	0.628
SVM	0.679	0.678	0.679	0.678
BERT-64-32	0.668	0.65	0.725	0.685
BERT-128-32	0.694	0.69	0.703	0.696
BERT-256-32	0.692	0.677	0.735	0.705
BERT-320-32	0.707	0.693	0.743	0.717
BERT-384-16	0.683	0.673	0.711	0.691
BERT-512-8	0.697	0.709	0.666	0.687

**Table 8:** Accuracy comparison between the proposed model and Bilal and Almazroi [6] models.

Classifier	Accuracy of the Proposed Model	Accuracy of the Bilal and Almazroi Models [6]	Difference
KNN	0.855	0.596	26%
NB	0.911	0.617	29%
SVM	0.946	0.679	27%
BERT-64-32	0.927	0.668	26%
BERT-128-32	0.953	0.694	26%
BERT-256-32	0.966	0.692	27%
BERT-320-32	0.968	0.707	26%
BERT-384-16	0.971	0.683	29%
BERT-512-8	0.97	0.697	27%
BERT-384-32	0.973	0	97%
BERT-512-32	0.968	0	97%

**Table 9:** F1 score comparison between the proposed model and Bilal and Almazroi [6] models.

Classifier	F1 Score of th Proposed Model	F1 Score of the Bilal and Almazroi Models [6]	Difference
KNN	0.853	0.591	26%
NB	0.91	0.628	28%
SVM	0.945	0.678	27%
BERT-64-32	0.926	0.685	24%
BERT-128-32	0.952	0.696	26%
BERT-256-32	0.965	0.705	26%
BERT-320-32	0.969	0.717	25%
BERT-384-16	0.971	0.691	28%
BERT-512-8	0.97	0.687	28%
BERT-384-32	0.973	0	97%
BERT-512-32	0.967	0	97%

**Table 10:** Comparison between the proposed model and previous studies on the Yelp dataset.

Paper	Model	Accuracy	Yelp Dataset
Mutinda et al. [20]	BERT	84.00%	Binary label
Mutinda et al. [20]	LoBERT (BERT+ CNN)	88.20%	Binary label
Bilal and Almazroi [6]	BERT-320-32	71.7%	Binary label
The Proposed model	BERT-320-32	96.9%	Binary label
The Proposed model	BERT-384-32	97.3%	Binary label

## Data Availability

The Yelp review dataset, which is publicly available, is utilized in this manuscript. https://huggingface.co/datasets/yelp_review_full and https://www.yelp.com/dataset/download.
